# Nature, Signs, and Treatment of Childbed Fever

**Published:** 1855-01

**Authors:** 


**ON THE NATURE, SIGNS, AND TREATMENT OF CHILDBED FEVERS.** By CHAS. D. MEIGS, M. D., Prof. of Midwifery and Diseases of Women and Children, Jefferson Medical College, Philadelphia.

Dr. Meigs is one of the most laborious men connected with medical science in the country, and his labors have enriched it. This last offering increases his claims upon the gratitude of the profession, and adds largely to his fame as a teacher and a writer. He approaches the discussion of a subject as if he had examined it to to the bottom, and when he has arrived at what he believes to be correct conclusions, he avows them without hesitation. His style and his mode of conveying his thoughts are peculiarly his own, and is responsible, and it would appear that he is held to a rigid accountability. We have no particular fault to find with either, as we have yet found no difficulty in comprehending his meaning.—On the contrary, beside his vigorous style, there is something fresh and enchaining in it. There is no difficulty in understanding his propositions, and his illustrations are clear and forcible. If the first are true, and the second fair and just, then this is all we care for or ask.

We take this occasion to return our thanks for the favor of this last work, which was a present to us in person from the venerable and justly celebrated author.

[*By Blanchard & Lea, Phila.*

